# Identification of MicroRNAs Regulating the Developmental Pathways of Bone Marrow Derived Mast Cells

**DOI:** 10.1371/journal.pone.0098139

**Published:** 2014-05-21

**Authors:** Yang Xiang, Fiona Eyers, Ian G. Young, Helene F. Rosenberg, Paul S. Foster, Ming Yang

**Affiliations:** 1 Centre for Asthma and Respiratory Disease, School of Biomedical Sciences and Pharmacy, Faculty of Health, University of Newcastle and Hunter Medical Research Institute, Callaghan, New South Wales, Australia; 2 Department of Physiology, Xiangya School of Medicine, Central South University, Changsha, Hunan, People's Republic of China; 3 Department of Molecular Bioscience, John Curtin School of Medical Research, Australian National University, Canberra, Australian Capital Territory, Australia; 4 Inflammation Immunobiology Section, Laboratory of Allergic Diseases, National Institute of Allergy and Infectious Diseases, National Institutes of Health, Bethesda, Maryland, United States of America; Ecole Normale Superieure de Lyon, France

## Abstract

**Background:**

MicroRNAs (miRNAs) play important roles in leukocyte differentiation, although those utilised for specific programs and key functions remain incompletely characterised. As a global approach to gain insights into the potential regulatory role of miRNA in mast cell differentiation we characterised expression in BM cultures from the initiation of differentiation. In cultures enriched in differentiating mast cells we characterised miRNA expression and identified miRNA targeting the mRNA of putative factors involved in differentiation pathways and cellular identity. Detailed pathway analysis identified a unique miRNA network that is intimately linked to the mast cell differentiation program.

**Methodology/Principal Findings:**

We identified 86 unique miRNAs with expression patterns that were up- or down- regulated at 5-fold or more during bone marrow derived mast cells (BMMC) development. By employing TargetScan and MeSH databases, we identified 524 transcripts involved in 30 canonical pathways as potentially regulated by these specific 86 miRNAs. Furthermore, by applying miRanda and IPA analyses, we predict that 7 specific miRNAs of this group are directly associated with the expression of c-Kit and FcεRIα and likewise, that 18 miRNAs promote expression of Mitf, GATA1 and c/EBPα three core transcription factors that direct mast cell differentiation. Furthermore, we have identified 11 miRNAs that may regulate the expression of STATs-3, -5a/b, GATA2 and GATA3 during differentiation, along with 13 miRNAs that target transcripts encoding Ndst2, mMCP4 and mMCP6 and thus may regulate biosynthesis of mast cell secretory mediators.

**Conclusions/Significance:**

This investigation characterises changes in miRNA expression in whole BM cultures during the differentiation of mast cells and predicts functional links between miRNAs and their target mRNAs for the regulation of development. This information provides an important resource for further investigations of the contributions of miRNAs to mast cell differentiation and function.

## Introduction

Mast cells (MCs) are vital innate immune cells that are well-known for their roles in the pathogenesis of immunoglobulin E (IgE)-mediated disorders [1], while aberrant generation of MCs can lead to disorders such as urticaria pigmentosa, systemic mastocytosis and mast cell activation syndrome [2]–[4]. There is also increasing recognition of the importance of MCs to both innate and adaptive host defence against bacterial and parasitic infection [5], [6]. Thus, gaining a better understanding of pathways that control MC differentiation will provide important insights into the developmental programs of MCs in both health and in disease states.

MCs are derived from pluripotent progenitors that originate in bone marrow and that mature in most tissues but are particularly enriched in the skin, tongue and in the mucosal layer of lung and gastrointestinal tracts, under the control of specific growth and differentiation factors [7]. Expansion of MCs predominantly and synergistically relies on stem cell factor (SCF) and interleukin (IL)-3 [8]; both human and murine MCs express receptors for these two cytokines [9], [10]. Interestingly, IL-3 alone cannot generate MCs, but this cytokine greatly enhances the numbers of differentiated mast cells in tissue culture and in vivo [10], [11]. By contrast, mice with SCF receptor (c-Kit) mutations (Kit^w/wv^ and Kit^w-sh/w-sh^ mice) have drastically reduced c-Kit/SCF signaling and demonstrate a profound reduction in MCs [12], [13]. Likewise, clinical evidence suggests that an activating mutation in the c-Kit gene is strongly associated with systemic mastocytosis [14].

Although SCF and IL-3 drive the development of MCs, the transcription factors and pathways that specifically control differentiation have not been fully characterised. Currently, microphthalmia-associated transcription factor (Mitf) is considered as a central mediator of MC differentiation; Mitf is highly expressed in both progenitor and fully differentiated MCs, while notably absent in basophils [15]. Furthermore, accumulating evidence suggests that the interplay between four co-re-regulatory factors (e.g. the up-regulation of Mitf, GATA2 and PU.1 and the down-regulation of CCAAT/enhancer binding protein α (C/EBPα)) primarily regulates the development of the MC lineage [16]–[18]. However, other intracellular signaling events such as signal transduction and transcription (STAT) proteins, GATAs 1 and 3 and epigenetic regulators may also contribute to the differentiation program [1], [18].

MicroRNAs (miRNAs) are emerging as regulators of the differentiation and proliferation of MCs [19]. MiRNAs are short non-coding RNAs that regulate gene expression post-transcriptionally by binding to the 3′ untranslated region of target mRNAs, thereby repressing protein production or destabilising target transcripts [20]–[22]. The numerous roles of these small molecules in the regulation of development, metabolism, organ function, and disease pathogenesis have been documented extensively [20]–[22]. By contrast, much less is known about the miRNA networks that control the differentiation and function of specific leukocyte subsets. The aim of this study is to explore miRNA expression profiling and analysis *in silico* in order to improve our understanding of the factors that regulate MC growth and differentiation. As a first approach, we have characterised the expression of miRNA in whole BM cultures from the initiation of MC differentiation to globally characterise the dynamics of miRNA expression in this cellular milieu. The miRNA expression profile in cultures enriched in differentiating MCs was determined and correlated to expression of molecules known to be critical for mast cell development by stringent statistical and pathways analysis. With these approaches, we report a unique miRNA network that is closely linked to the MC differentiation program. Our investigation provides novel information on the miRNA networks interacting with one another during MC differentiation and demonstrates how specific miRNA may potentially regulate the crucial differentiation factors and biosynthesis of MC mediators.

## Methods

### Animals

Specific pathogen free BALB/c mice (6–8 weeks) were obtained from the animal services unit of the University of Newcastle. All experiments were performed with approval from the Animal Ethics Committee of the University of Newcastle (Permit Number: A-2010-136). All surgery was performed under sodium pentobarbital anesthesia, and all efforts were made to minimize suffering.

### Culture and identification of bone marrow derived mast cells

Bone marrow derived mast cells (BMMCs) were differentiated and examined as previously described, with slight modifications [23]. Briefly, mouse femurs were flushed with 5 ml ice cold HBSS through a 70 µm cell strainer. After lysis of red blood cells and washing with PBS, BMMCs were cultured for 6 weeks in RPMI 1640 medium (Invitrogen) supplemented with 10% FCS, 4 mM glutamine, 100 U/ml penicillin, 100 µg/ml streptomycin, 25 mM hydroxyethyl piperazineethanesulfonic acid, 1 mM sodium pyruvate, and 50 µM 2-mercaptoethanol, 15 ng/ml SCF (Peprotech) and 30 ng/ml IL-3 (Peprotech) at a starting concentration of 1×10^6^ cells/ml and cultured at 37°C in a humidified atmosphere of 5% CO_2_ and 95% air. Culture medium was replaced twice per week for 6 weeks and the cell concentration was maintained at 1×10^6^ cells/ml. 2.5×10^7^ cultured cells were harvested every week for miRNA microarray and qPCR. The purity of BMMCs was determined by using toluidine blue staining of cytospins (1×10^6^ BMMCs per slide) and flow cytometry (See Method S1).

### MicroRNA microarray

Total RNA was extracted from bone marrow cells and BMMC cultures from week 2 to week 6 using TRIzol reagents (Life Technologies), and miRNA microarray was performed as previously reported [24]. Briefly, the Agilent spike-In control was added to 100 ng RNA, which was dephosphorylated by incubating the samples at 37°C for 30 minutes followed by ligation of Cy3 using the Complete Labelling and Hybridisation c-Kit (Agilent). Following ligation and drying, the Cy3-labelled RNA samples were hybridized for 20 h at 55°C to Agilent 8×15K mouse microRNA array slides (AMADID 21828), which included 627 mouse miRNA and 39 mouse viral miRNA from the Sanger database 12.0. After washing with Agilent gene expression wash buffers, the hybridized microarrays were scanned on a High Resolution C scanner (Agilent). Data were extracted from scanned microarrays using Feature Extraction software (version 10.7.3.1). The fluorescence index of each miRNA at different time-points was further normalized to that of the respective miRNAs in control group (isolated bone marrow cells). The normalized microarray data were managed and analyzed by GeneSpring (Agilent). MicroArray data have been deposited into ArrayExpress (http://www.ebi.ac.uk/arrayexpress/). The accession number is E-MTAB-2443.

### Quantification of microRNA and Messenger RNA

Quantitative polymerase chain reactions (qPCR) were performed as previously described (also See Method S1) [24]. Primer sequences are shown in **Table S1**.

### MicroRNA target analysis

For prediction of target genes of differentially expressed miRNAs, we first used TargetScan 6.1 (http://www.targetscan.org/) to identify potential targets. MeSH database (http://www.nlm.nih.gov/mesh/meshhome.html) was then employed to identify the molecules relevant to MC biology by exact syntax matching. MiRanda (http://www.microrna.org/) was also used to refine the predicted targets. Ingenuity Pathways Analysis (Ingenuity Systems, Redwood City, CA) software was employed so as to define canonical signaling pathways containing the miRNA-associated MC-relevant molecules, and to establish the connection between miRNAs and their respective predicted targets.

### Statistical analysis

An initial one-way ANOVA was followed by appropriate comparisons to test for differences between means of groups. Values are reported as the means ± SEM for each experimental group. The number of samples at each time-point ranged from 4 to 6. Differences in means were considered significant if p was <0.05.

## Results

### Generation of BMMC

**Figure 1:**
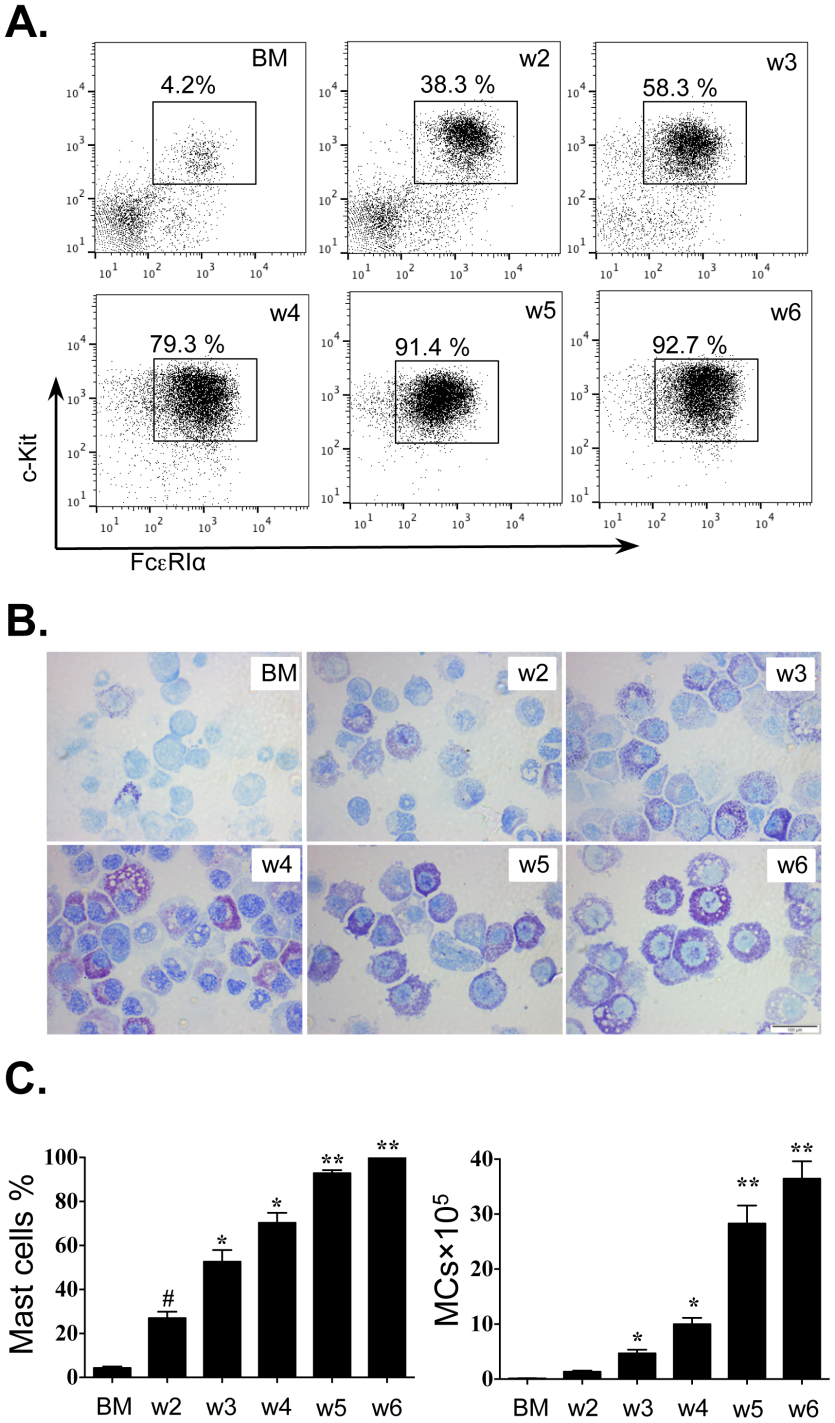
Expansion of BMMCs *ex vivo*. Bone marrow cells from BALB/c mice were cultured for 6 weeks as described in the Methods. MCs were identified by (A) flow cytometry (c-Kit^+^FcεRIα^+^ cells) and (B, C) toluidine blue staining. BM, bone marrow; w2 – w6, weeks 2–6; original magnification of 100×. Values are presented as mean ±SEM (n = 4∼6), **P<0.05 (w5 or w6 vs. other groups). *P<0.05 (w3 or w4 vs. BM and w2). #P<0.05 (w2 vs. BM).

Isolated bone marrow cells from BALB/c mice were cultured for 6 weeks in the presence of SCF and IL-3. From week 2 to week 6, the percentage of BMMC (c-Kit^+^ FcεRI^+^) in the culture was determined first by flow cytometry. The morphological features, the percentages and numbers of BMMCs were also determined by toluidine blue staining of cytospin preparations. The percentage c-Kit^+^FcεRI^+^cells increased from 4.2% to 92.7% in BMMC culture at week 6 (**Fig. 1A**). We observed a parallel increase in toluidine-blue positive cells (5.34±1.9% to 99.0±0.03%; 2.70±0.21×10^5^ to 36.5±3.2×10^5^ cells/mL; **Figs. 1B and C**, respectively.)

### Distinct miRNA expression profile during BMMC development

**Figure 2:**
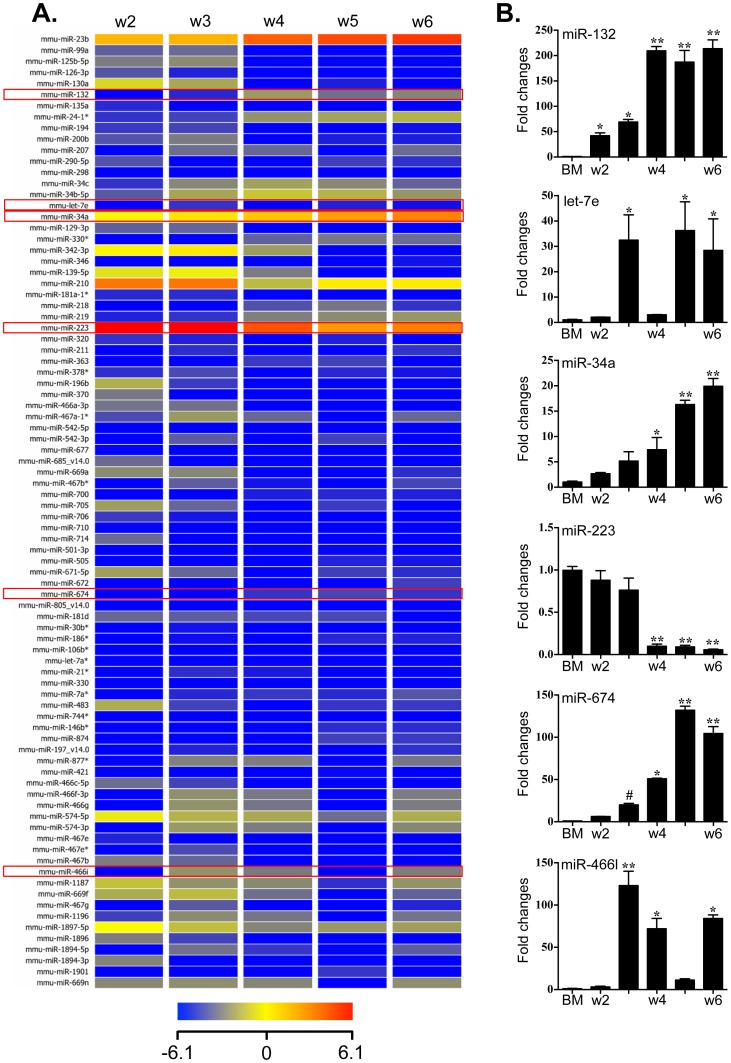
Profiling the expression of miRNAs during BMMC differentiation. RNA was isolated from cultured bone marrow cells from week 2 to week 6 in the presence of SCF and IL-3, representing three independent mast cells cultures. (A) Heat map representation documenting expression levels of 86 miRNAs identified as up- or down-regulated 5-fold or more. The fluorescence index of each miRNA at different time-points was further normalized to that of the respective miRNAs in control group (isolated bone marrow cells). The normalized microarray data were managed and analyzed by GeneSpring (Agilent). Scale ranges from a signal value of -6.1(blue) to +6.1(red). (B) Confirmation of miRNA array expression by Taqman quantitative PCR. 6 miRNAs (miRNA −132, −34a, −223, −466l, −674 and let-7e) were selected to verify the expression profile of the miRNA array. Values are presented as mean ±SEM (n = 4∼6), **P<0.001 (vs. BM). *P<0.01 (vs. BM). #P<0.05 (w3 vs. w2).

Given the broad involvement of miRNAs in regulating post-transcriptional gene expression, we proceeded to determine the miRNA profile of BMMC cultures from week 2 to week 6. Total RNA was isolated from homogenised cells and screened as described in Methods. We identified eighty-six (86) miRNAs characterised with 5-fold differential expression during BMMC development; among these, we document 45 miRNAs with increased expression and 41 with decreased expression, respectively (**Fig. 2** **A**). Detailed information describing these miRNAs is included in **Table S2 in File S1**. We validated the differential expression of six (6) of the 86 miRNAs with qPCR (**Fig. 2** **B**). Among the miRNAs selected for validation, miR-223 has a central role myeloid cell development [25]. The other miRNAs have been linked to cell development, inflammation and disease [26]–[30]. The expression profiles defined for these six miRNAs by qPCR were consistent with the expression patterns documented on the microarray.

### Analysis of BMMC related molecules and canonical pathways potentially regulated by the miRNAs

**Figure 3:**
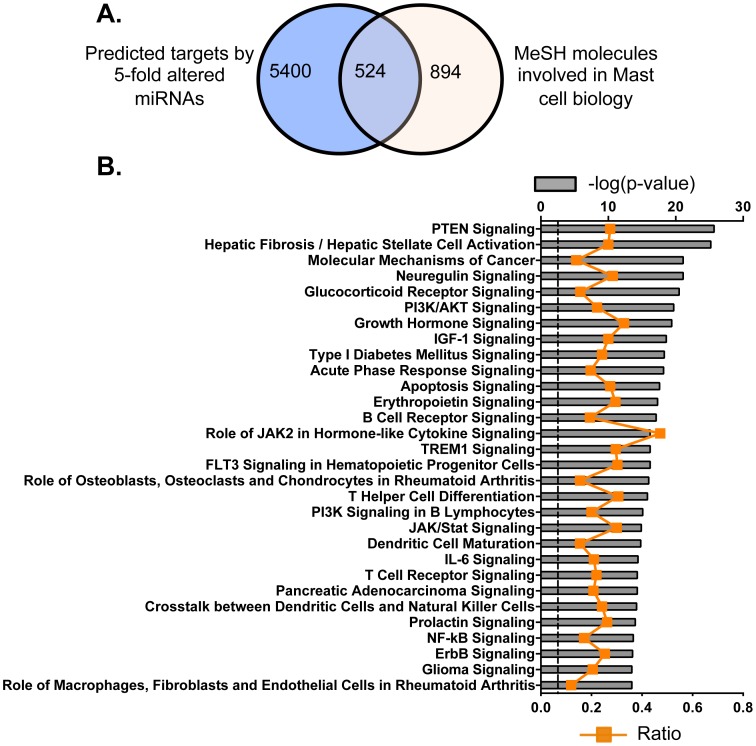
Potential molecules and top canonical pathways that were predicted and targeted by the miRNAs identified as differentially regulated during BMMC differentiation. (A) Target prediction by TargetScan database (http://www.targetscan.org/) was established on sequence data complementarity to target 3′UTR sites. Target molecules, associated with MC biology, were identified by exact syntax matching in the MeSH database (http://www.nlm.nih.gov/MeSH/MeSHhome.html). (B) Top 30 canonical pathways that include selected 524 molecules as identified by IPA. The significance of association between selected genes and canonical pathway was evaluated by a right-tailed Fisher's exact test (grey bars, upper y-axis). Ratios referring to the proportion of selected genes from a pathway related to the total number of molecules that make up that particular pathway were also displayed (line graph, bottom y-axis).

In an effort to elucidate targets and pathways regulated by the 86 differentially-expressed miRNAs, we proceeded to identify putative mRNA targets using TargetScan (version 6.1). TargetScan is a search engine that predicts targets for miRNA-mRNA interactions in eukaryotes; the algorithms search for 8- and 7-mer sites located in the 3′-UTR regions of mRNAs which share sequence homology with query miRNA sequences [31]; predictions are then ranked by their probability of targeting the mRNA transcript [32]. We used a 95^th^ percentile cutoff to predict the potential targets. With this method, we identified 5924 mRNA transcripts that could be targeted by the 86 miRNAs (**Fig. 3** **A**). The target mRNAs were further refined by searching the MeSH database, in an effort to link gene expression data with known MC-related pathways, diseases and phenotypes. There were 1418 mRNAs identified by MeSH database as containing the exact terms “mast cell, SCF, IL-3 and tryptase” with a link to at least one PubMed-affiliated reference. We then plotted differences in distribution of mRNAs predicted by TargetScan and MeSH databases, and found that, of the original 5924 mRNA transcripts, we could focus on 524 that were targeted by both search methods (**Table S3 in File S1**).

These 524 MC mRNAs were then organized and classified with the assistance of Ingenuity Pathways Analysis (IPA); the top 30 canonical pathways were listed (**Fig. 3** **B; Table S4 in File S1**). Two-hundred and three (203) of the aforementioned molecules (38.7% of the 524 MC-associated transcripts) were included within these top 30 canonical pathways. Although many of the pathways are commonly involved in cell death and survival or proinflammatory activity, these pathways also include those that contribute to mast cell differentiation and function. For example, Phosphatase and Tensin Homolog (PTEN) regulates the proliferation MC; PTEN deficiency results in a mastocytosis-like proliferative disease [33]. Likewise, activation of Glucocorticoid Receptor signaling suppresses both proliferation and degranulation of MCs [34], and PI3/AKT signaling is critical for the maturation and pro-inflammatory factor production by MC [35]. Pathways such as Growth Hormone, IGF-1, Apoptosis and Role of JAK2 in Hormone-Like Cytokine may regulate MC survival and death [8]. FLT3 Signaling in Hematopoietic Progenitor Cells, Erythropoietin, IL-6 and ErbB signaling may play central roles in differentiation of haematopoietic progenitor cells to MC specific lineage [36]. Well-known inflammatory pathways such as Acute Phase Response and NF-kB likewise regulate the production of proinflammatory factors in MCs, and NF-kB can also modulate MC proliferation [37], [38]. TREM1 may play a central role in MC responses to bacterial invasion [37]. Interestingly, activation of MCs has long been recognized as an important component of the pathogenesis of rheumatoid arthritis [39]; the two signaling pathways, Role of Osteoblast/Osteoclast and Chondrocytes in Rheumatoid Arthritis and Role of Macrophages/Fibroblast and Endothelial Cell in Rheumatoid Arthritis, may feature components of this role. Taken together, these data suggest important roles for miRNAs in the regulation of trafficking, biological function and cell death/cell survival of BMMCs.

### Multiple miRNAs are linked to the expression of c-Kit and FcεRI but not IL-3Rα

**Figure 4:**
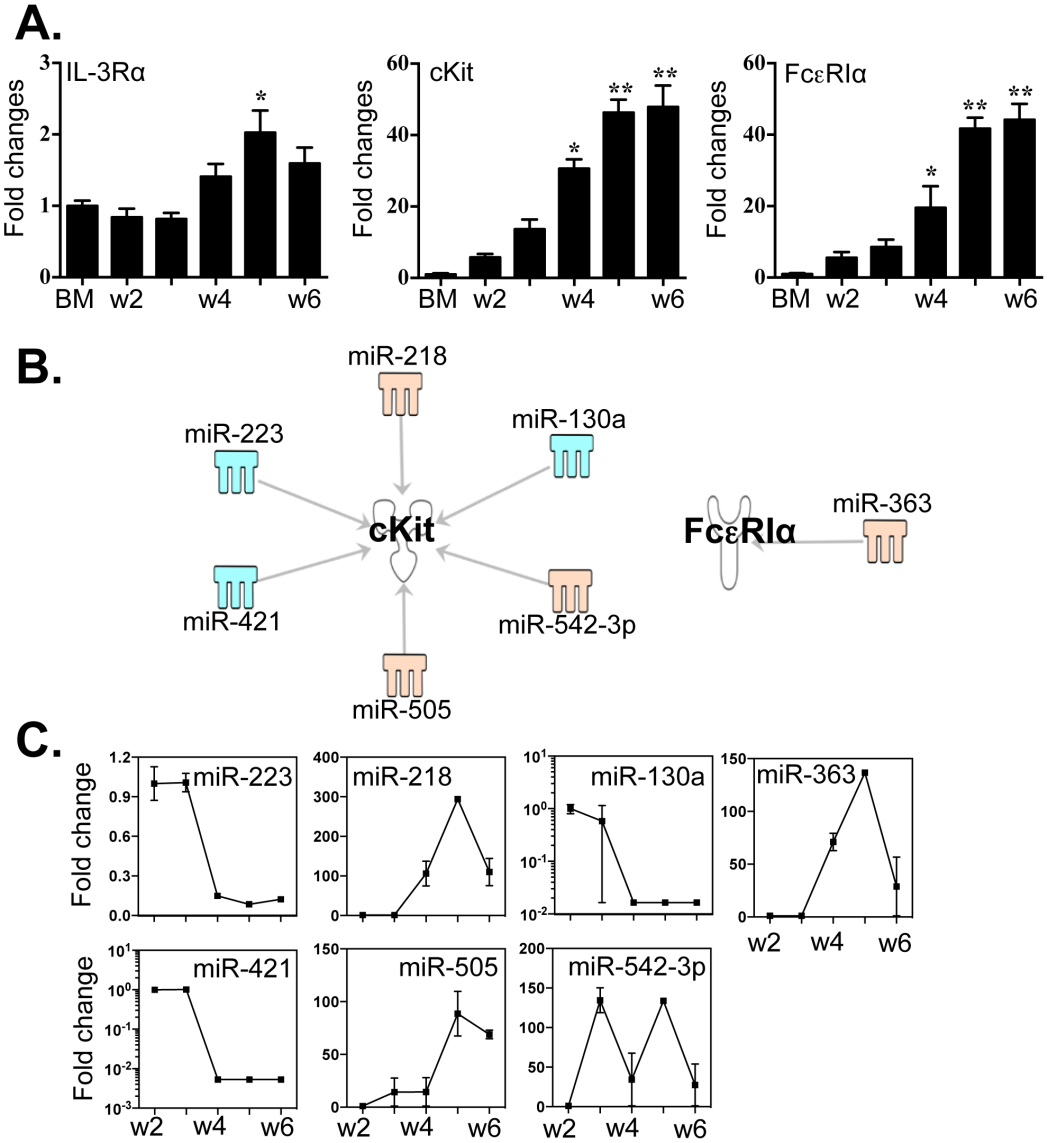
Expression levels of IL-3Rα, c-Kit and FcεRIα correlated with the expression of miRNAs that might target these transcripts. Data represent findings from three independent mast cell cultures. (A) Expression levels of IL-3Rα, c-Kit and FcεRIα were determined by qPCR. (B) Potential miRNAs targeting the 3′-UTR of c-Kit and FcεRIα were identified by TargetScan, MiRanda database and IPA ingenuity system. Blue represents decreased expression of miRNAs, whereas yellow is for increased expressed miRNAs. (C) The fold changes of potential regulating miRNAs were calculated based on the fluorescence index of each miRNA at different time-points, after normalization to that of the respective miRNAs in the control group (isolated bone marrow cells). Values are presented as mean ±SEM (n = 4∼6), **P<0.001 (vs. BM). *P<0.01 (vs. BM).

IL-3Rα, c-Kit and FcεRI are critical signature receptors of MCs; we examined the expression of these molecules in BMMC cultures from week 2 to week 6 by qPCR (**Fig. 4** **A**). Relative expression of transcripts encoding IL-3Rα underwent a small but statistically significant increase between week 2 and week 5. By contrast, expression levels of c-Kit and FcεRIα increased markedly (greater than 40-fold over baseline by week 6). We used IPA and the miRanda database in order to determine whether any of the 86 differentially expressed miRNAs (Fig. 2 A) might have binding sequences that could target the 3′-UTRs of any of these specific transcripts. Interestingly, none of the aforementioned 86 miRNAs has any clear potential to regulate IL-3Rα. However, the c-Kit transcript is linked to 6 specific miRNAs, of which 3 (miR −218, −505 and -542-3p) undergo an increase and 3 (miR -130a, -223 and -421) a decrease (**Fig. 4** **B** and **C**) during BMMC differentiation in culture. The transcript encoding FcεRI may be targeted by miR-363. The 3′-UTR binding sites of these miRNAs are included in **Table S5 in File S2**.

### Critical MC transcriptional regulators targeted by distinct sets of miRNAs

**Figure 5:**
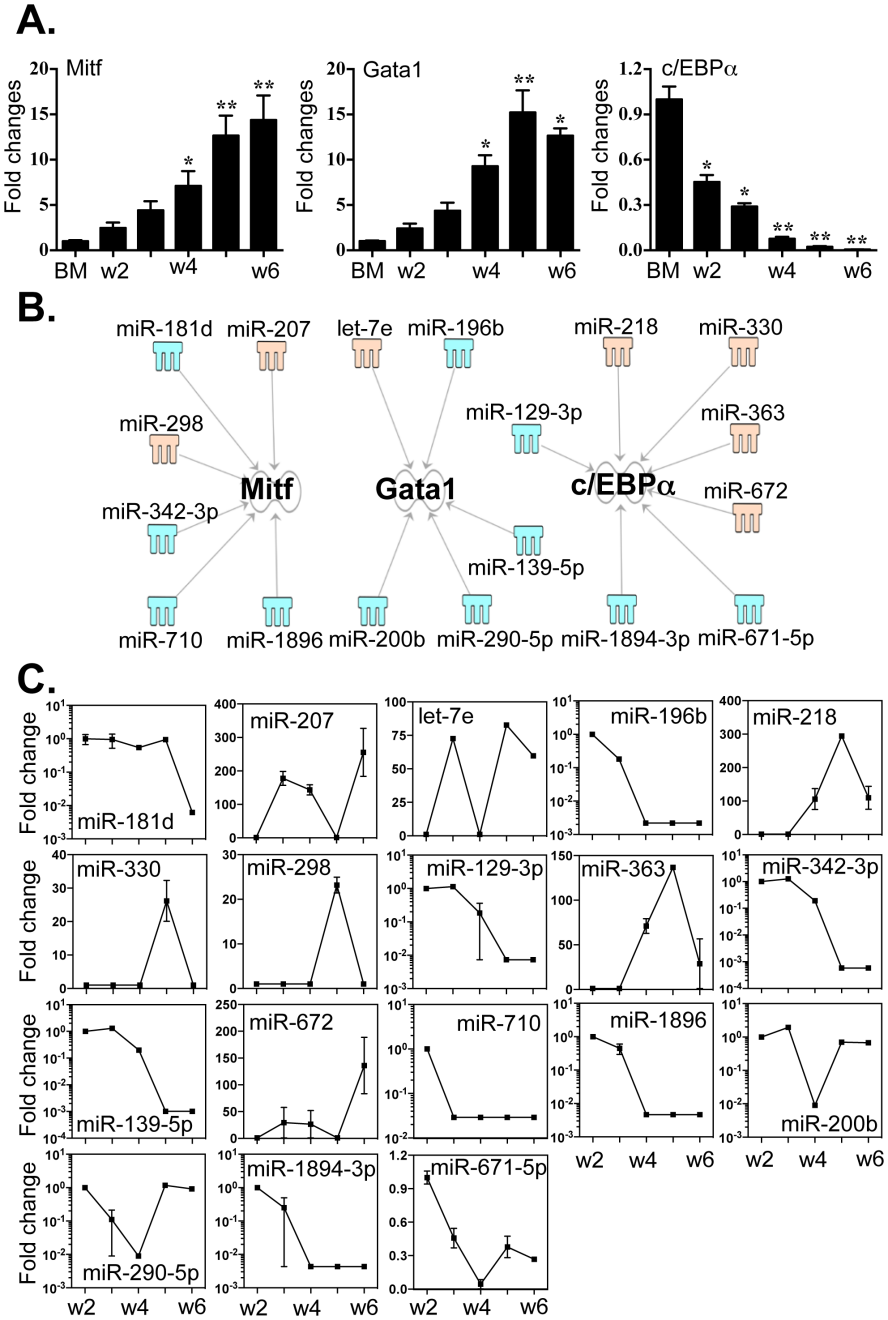
Expression of Mitf, GATA1 and c/EBPα correlated to the expression levels of miRNAs that potentially target these transcripts. (A). Expression levels of Mitf, GATA1 and c/EBPα were determined by qPCR. (B) Potential miRNAs targeting the 3′-UTR of Mitf, GATA1 and c/EBPα were identified by TargetScan and Miranda database and IPA ingenuity system. Blue represents decreased expression of miRNAs, whereas yellow is for increased expressed miRNAs. (C). The fold changes of potential regulating miRNAs were calculated based on the fluorescence index of each miRNA at different time-points, after normalization to that of the respective miRNAs in the control group (isolated bone marrow cells). Values are presented as mean ±SEM (n = 4∼6), **P<0.001 (vs. BM). *P<0.01 (vs. BM).

The fate of BMMCs is critically determined by a coordinated action by transcription factors Mitf, GATA1, c/EBPα and PU.1 [17], [18]. We evaluated the differential expression of these factors by qPCR and correlated these patterns to those of the 86 differentially expressed miRNAs (Fig. 2 A). As previously reported [15], [16], expression levels of Mitf and GATA1 were significantly elevated during differentiation of BMMCs while that of c/EBPα decreased dramatically (**Fig. 5** **A**). PU.1 expression did not change, although it was highly expressed overall in BMMC culture (data not shown). Using IPA and miRanda, we identified three sets of miRNAs that have to potential to regulate the expression of these four MC transcriptional regulators (**Fig. 5** **B**). We correlated the expression levels of specific miRNAs to Mitf (miR −181d, −207, −298, −342-3p, −710 and −1896), GATA1 (let-7e, miR −196b, 139–5p, −200b and −290–5p) and c/EBPα (miR −129–3p, −218, −330, −363, −672, 1894–3p and 671-5p), but interestingly, found no miRNAs with the capacity to target PU.1 (**Fig. 5** **C**). The 3′-UTR binding sites of miRNAs that target all three of these mRNAs are shown in **Table S6 in File S2**. These findings likewise suggest that miRNAs have the potential for extensive and critical involvement in the regulation of MC differentiation.

### miRNAs connected to the expressions of STAT3, STAT5a, STAT5b, GATA2 and GATA3

**Figure 6:**
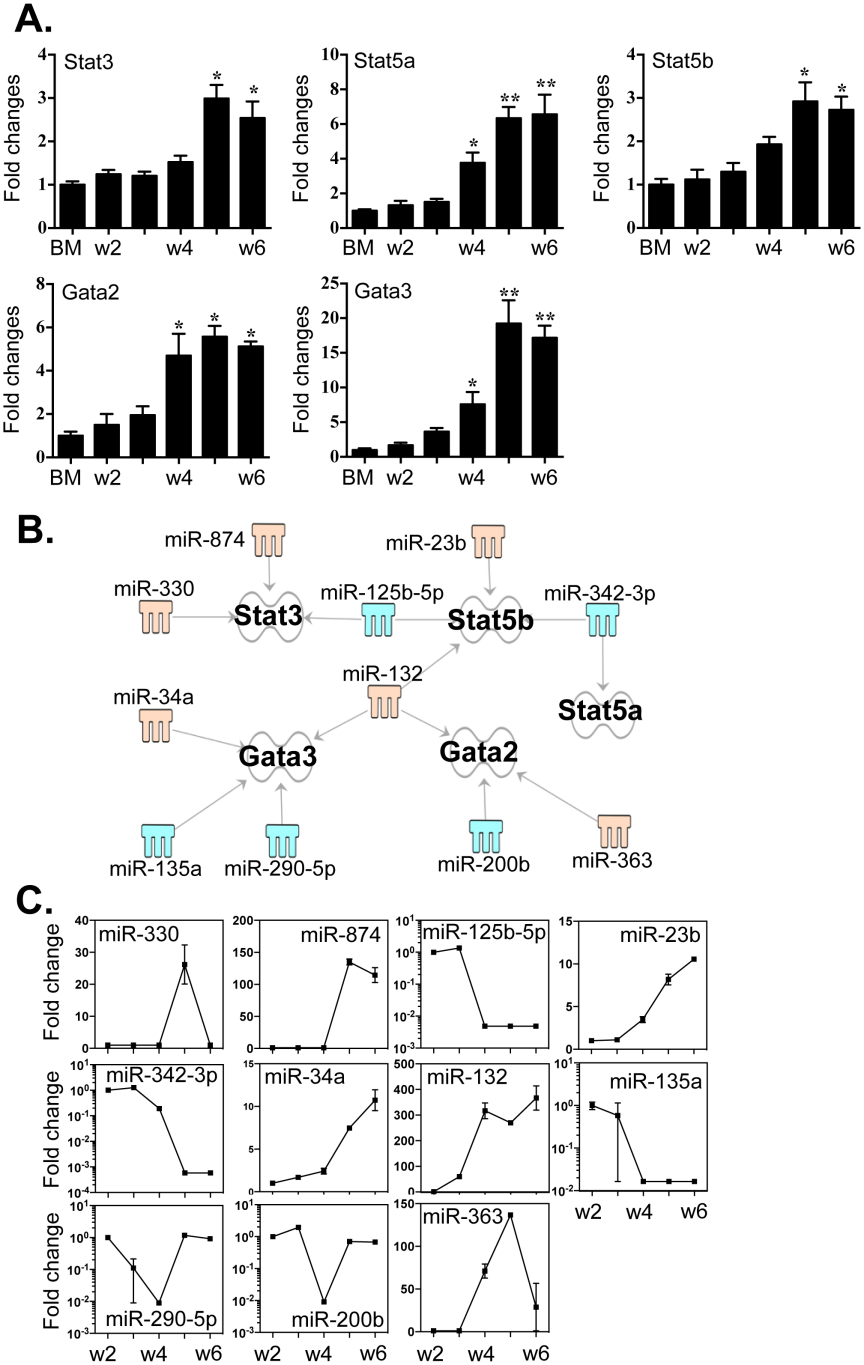
Expression of STAT3, STAT5a/b, GATA2 and GATA3 correlated to the expression of miRNAs that potentially target these transcripts. (A). Expression levels of STAT3, STAT5a/b, GATA2 and GATA3 were determined by qPCR. (B). Potential miRNAs targeting the 3′-UTR of STAT3, STAT5a/b, GATA2 and GATA3 were identified by TargetScan and MiRanda database and IPA ingenuity system. Blue represents decreased expression of miRNAs, whereas yellow is for increased expressed miRNAs. (C). The fold changes of potential regulating miRNAs were calculated based on the fluorescence index of each miRNA at different time-points, after normalization to that of the respective miRNAs in the control group (isolated bone marrow cells). Values are presented as mean ±SEM (n = 4∼6), **P<0.001 (vs. BM). *P<0.01 (vs. BM).

We proceeded to determine whether any of the 86 differentially-expressed miRNAs could target other factors that have been implicated in the regulation of MC differentiation. STAT3 and STAT5a/b play pivotal roles in transducing signals associated with SCF and IL-3 induction of MC differentiation [1]. The transcription factors GATA2 and GATA3 are also important in promoting differentiation of MCs from bone marrow-derived myeloid progenitors [1], [16]–[18]. We first determined the expression levels of STAT3, STAT5a, STAT5b, GATA2 and GATA3 during BMMC culture (**Fig. 6** **A**). All of these factors responded to culture conditions with increased expression from week 2 to week 6. Interestingly, the miRNAs that targeted these mRNA transcripts (**Fig. 6** **B**), further refined by IPA and miRanda analysis, mostly overlapped with those identified in Fig. 5B and Fig. 6B. The interactions between the 3′-UTRs of these five mRNAs and their associated miRNAs are presented in **Table S7 in File S2**. For STAT3, the two of the three associated miRNAs (miR −330 and −874) responded with increased expression and one (miR-125b-5p) decreased; for STAT5b, two miRNAs (miR −125b-5p and −342-3p) responded with decreased expression and one (miR-23b) increased (**Fig. 6** **C**). Decreased expression of miR-342-3p was also associated with increased levels of STAT5a, and we found that one factor, miRNA-132, may concurrently target STAT5b, GATA2 and GATA3. Notably, there is considerable overlap between the miRNAs that regulate the three core transcription factors (**Fig. 5**), and the aforementioned STATs and GATA2/GATA3 regulators (**Fig. 6**). The distinct role of these miRNAs in fine-tuning the expression of these molecules in MC differentiation remains to be explored.

### Association between miRNAs expression and major functional transcripts of BMMC

**Figure 7:**
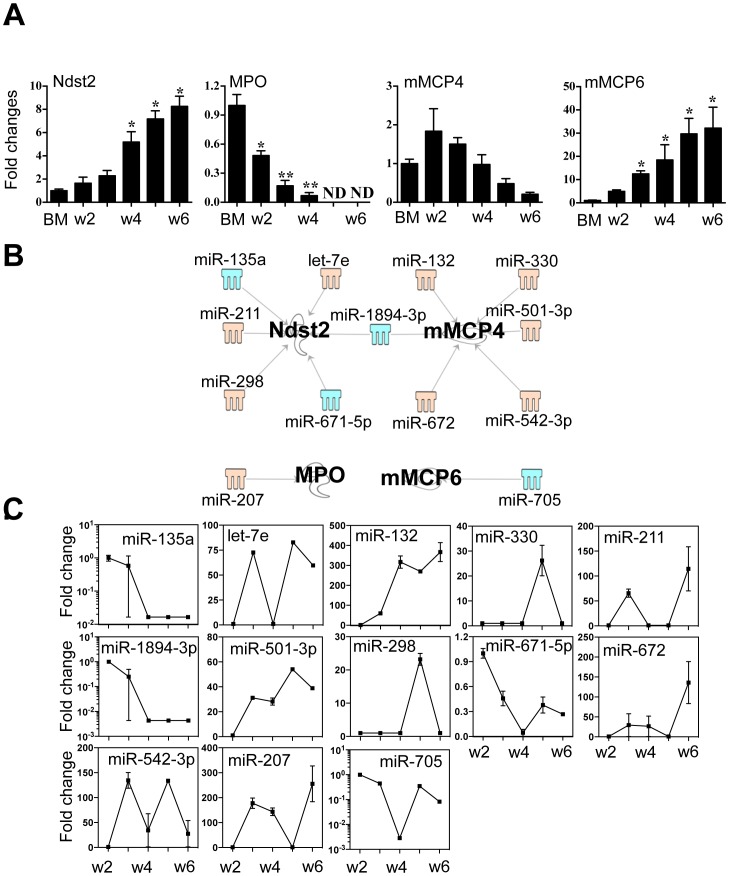
Expression of Ndst2, MPO, mMCP4 and mMCP6 correlated to the expression of miRNAs that potentially target these transcripts. (A) Expression levels of Ndst2, MPO, mMCP4 and mMCP6 were determined by qPCR. (B) Potential miRNAs targeting the 3′-UTR of Ndst2, MPO, mMCP4 and mMCP6 were identified by TargetScan and Miranda database and IPA ingenuity system. Blue represents decreased expression of miRNAs, whereas yellow is for increased expressed miRNAs. (C) The fold changes of potential regulating miRNAs were calculated based on the fluorescence index of each miRNA at different time-points, after normalization to that of the respective miRNAs in the control group (isolated bone marrow cells). Values are presented as mean ±SEM (n = 4∼6), **P<0.001 (vs. BM). *P<0.01 (vs. BM).

MC granules contain substantial amounts of heparin, histamine, MC-specific proteases (tryptase and chymase) and other unique biological factors [7]. N-deacetylase/N-sulfotransferase (Ndst) 2 is a crucial enzyme in the biosynthetic pathway of heparin and its expression is strongly linked to the process of MC maturation [40]. Likewise, histamine is synthesized by an enzymatic reaction catalyzed by L-histidine decarboxylase (HDC), while MC proteases are each encoded by the unique genes, mast cell protease (mMCP) −2, −4, −5, −6 and −7 [7]. We determined that transcripts encoding Ndst2, mMCP6 and mMCP7 were significantly up-regulated from week 2 to week 6, whereas the expression of myeloperoxidase (MPO, predominantly expressed in neutrophils) decreased to nearly undetectable levels at wk 6 (**Fig. 7** **A**). HDC was constantly expressed at high levels over the same interval (unpublished observation). Mouse MCP4 increased marginally at week 2 but was down-regulated to 21% of initial levels by week 6 (**Fig. 7** **A**). Ndst2 expression was linked to that of three miRNAs (let-7e, miR −211 and −207) that showed increased expression and three miRNAs (miR −135a, −671-5p and −1894-3p) with decreased expression (**Fig. 7** **B** and **C**), while mMCP4 was associated with the increased expression of 5 miRNAs (miR −132, −330, 501-3p, 542-3p and −672) and the decreased expression of miR-1894-3p (**Fig. 7** **B** and **C**). MPO and mMCP6, were targeted by miR-207 and miR-705 which had increased and decreased expression, respectively. The interaction between 3′-UTR of the four mRNAs and their associated miRNAs are presented in **Table S8 in File S2**. Interestingly, the sequence of miR-1893-3p suggests that it is likely to target both Ndst2 and mMCP4.

## Discussion

Previous studies have identified several miRNAs, including miR -132, miR-146a and miR-221, that play roles in regulating MC apoptosis, cell cycle regulation, growth and degranulation [41]–[43]. Monticelli and colleagues have used 181-miRNAs-Array to profile matured BMMC and identified 15 of them with detectable changes. However, their method can not efficiently discriminate between close miRNA paralogs [44]. As a more recent example, Lee and colleagues have shown that decreased expression of both miR-539 and miR-381 in association with c-Kit signaling promotes expression of the transcription factor, Mitf, which leads to MC proliferation [45]. Our present study was undertaken in order to provide a comprehensive examination of miRNA temporal expression during differentiation of BMMC in culture starting within the initiation of differentiation in cellular milieu of the BM, with more extensive and gene-specific Agilent Array containing 627 mouse miRNA and 39 mouse viral miRNA. Then by using detailed pathway analysis programs we have linked miRNAs expression in cultures enriched in differentiating mast cells with the regulation of transcripts that play critical roles in mast cell development. Toward this end, we have established a BMMC *ex vivo* culture system and we have determined the expression of characteristic receptors, functional factors and critical transcription regulators during the development. The profile of expressed miRNAs was determined over time and correlated to expression of molecules known to be crucial to MC development by stringent statistical and validated pathways analyses. Via these methods, we documented an original MC expression signature that included 86 independent miRNAs that were differentially regulated five-fold or more over baseline levels during the development of BMMC between week 2 and week 6.

MiRNAs play critical roles in maintaining gene expression at appropriate levels and in fine-tuning a wide range of biological and disease processes [20]–[22]. However, it remains difficult to associate miRNAs with their targets either directly or experimentally and likewise to predict specific biological functions. For example, Siemens and colleagues have determined that c-Kit is a direct target of miR-34a by using several human colorectal cancer cell lines [46]. Although miR-34a has two continuous 8-mer binding sites at the 3-UTR of human c-Kit, it may bind weakly to the respective region of mouse c-Kit as there are only two discontinuous 5-mer seed sequences. This suggests that miR-34a is unlikely to directly regulate the expression of c-Kit in mouse. Interestingly, Shin and colleagues have identified that miR-34a contributes to BMMC apoptosis by targeting Bcl-2 [47]. These observations may show that the potential targets of miRNAs can be rather distinctive and these small regulatory molecules play fairly distant regulatory roles in biological development and the progression of diseases in different species.

Here, we have employed a combinatorial approach, and we searched for targets for the 86 differentially-regulated miRNAs initially based on algorithms from TargetScan and further filtered the potential targets by searching MeSH database for published links to MC biology. From there, our findings were refined and categorized into networks that associated miRNAs with critical MC factors using miRanda and IPA. With this approach, we have identified 32 unique miRNAs that are most likely to be involved in regulating MC differentiation and function via targeting critical MC factors.

By cross-comparison with TargetScan and MeSH database, we generated a list of 524 transcripts that have the highest-likelihood of serving as targets for the 86 differentially-expressed miRNAs and linked to MC biology. By IPA, we showed that 39% of these molecules were included within the top 30 signal transduction pathways, among which were molecules controlling cell cycle, growth and death, and cell activation. For example, PTEN and PI3K/AKT signaling are closely paired and serve to balance one another in order to regulate cell proliferation. Impairment of PTEN signaling may lead to PI3K/AKT hyperreactivity, resulting reduced apoptosis and unchecked cell proliferation [33]. Furthermore, miRNA may also target other pathways such as growth hormone, IGF-1 and FLT3 signaling to control MC differentiation. JAK/STAT and NF-kB signal transduction pathways that are responsible for cell differentiation and are pro-inflammatory are also potentially targeted by the identified miRNAs.

By examining connectivity between predicted targets, we distinguished between closely-linked molecules that may operate as network hubs, with highly associated factors functioning as similar units. Among the 524 potential miRNA targets, c-Kit and FcεRIα are the well-known signature receptors of MC [1], [9], [10]. The expression of c-Kit and FcεRIα were increased almost 400-fold from day 0 through 6 weeks of culture (Fig 5. A). These results are consistent with the greatly expanded c-Kit^+^ FcεRIα^+^ BMMC population. Although SCF and IL-3 are used for 4 to 8 weeks in most *ex vivo* BMMC cultures [8]–[13], [23], we have observed that none of 86 miRNAs target the IL-3Rα chain transcripts. However, transcripts for this receptor were only slightly increased during culture and levels were high prior to induction of differentiation indicating that this system was already functional (data not shown). Taken together, these results suggest that the IL-3 signaling pathway may be involved more broadly in myeloid differentiation and its contributions are not necessarily limited to MC maturation. By contrast, the interactions between SCF and c-Kit are essential for MC development; deleting c-Kit in a mouse model results in the deficiency of mast cells and elimination of IgE-mediated responses [12], [13]. Six miRNAs were closely associated to c-Kit expression during MC differentiation; miR −218, −421 and −542-3p recognize multiple sites in the 3′-UTR (**Table S4 in File S1**), suggesting that these miRNAs are critical for MC function. Furthermore, it appears that the expression of FcεRIα is only regulated by miR-363 from this dataset.

As per current models, cooperation between several transcriptional regulators determines the differentiation and function of mast cells [1], [16]–[18]. Our results suggest that three core transcriptional factors, Mitf, GATA1 and c/EBPα are differentially regulated by distinct groups of miRNAs (Fig. 6). The level of PU.1, which is another core regulator, did not change during BMMC culture, but levels of transcript remained high throughout (data not shown). Of note, there are other transcriptional factors involved in MC differentiation, such as STAT3, STAT5a/b and GATA2/3 [1], [16]–[18]. Indeed, appropriate activation of these transcriptional pathways in cell growth, differentiation, survival, chemotaxis and cytokine production is essential for MC growth and differentiation [1], [16]–[18]. Unlike the discrete connection between the distinct groups of miRNAs and Mitf, GATA1 and c/EBPα, the relationship between the STAT and GATA transcripts and miRNAs is more difficult to define (Fig. 7). This is partly because these proteins are directly activated by SCF and IL-3, and partly because that the activation of these transcription factors is largely regulated by their phosphorylation, rather than directly by increased protein expression. Nevertheless, it is noteworthy that miR-342-3p -with almost 1000-fold decreased expression- still targets STAT5a, STAT5b and Mitf. Furthermore, miR-125b-5p, that was decreased approximately 200 fold, is associated with the increased expression of STAT3 and STAT5b transcripts. This suggests that the down-regulation of the two aforementioned miRNAs potentially play critical roles in MC differentiation, particularly in the early phases of lineage commitment. On the contrary, miR-132 was significantly increased, but its potential targets (e.g. STAT5b and GATA2/3) were also increased. These findings suggest that miR-132 acts as an important checkpoint to maintain the expression of these transcriptional regulators at appropriate levels. Interestingly, miR-132 may also control the expression of the mast cell protease, mMCP-4, suggesting the broader roles for this miRNA in the MC biology.

Finally, to explore the connections between miRNAs and MC secretory function, we examined associations between selected miRNAs and the 3′UTRs of major MC secretory mediators. Among the thirteen miRNAs identified, interestingly, three miRNAs (miR −211, −501-3p and −705) were only linked to Ndst2, mMCP4 and mMCP6, respectively (Fig. 7). Not only with selected MC secretory mediators, seven of aforementioned 13 miRNAs (let-7e, miR −207, −298, −330, −671-5p, −672 and −1894-3p) were also associated with three core MC transcriptional regulators (Mitf, GATA1 and c/EBPα) (Figs. 6 and 8). Collectively, our data suggest that the molecular mechanisms controlling MC differentiation and function are closely interwoven where sets of miRNA play central roles.

Taken together, we present a model of BMMC differentiation via which we can examine expression patterns of miRNAs. We have analysed these patterns and identified miRNAs that may play pivotal roles in driving MC maturation and regulating MC function. However, to understand how these selected miRNAs interact with their targets, functional studies with miRNA targeting, knock-in and knock-down models are required. Nevertheless, we are able to demonstrate that these selected miRNAs can be grouped as networks in order to guide bone marrow cells toward MC development in cooperation with cytokine signaling. Furthermore, there are also a number of intracellular signaling pathways that may be regulated by these miRNAs. Manipulating the expression of these miRNAs may promote further understanding of the underlying mechanisms that are critical for MC differentiation and function, and ultimately provide therapeutic targets for treating MC-associated diseases.

## Supporting Information

Method S1 **Toluidine blue staining, flow cytometry and miRNA/mRNA qPCR were described.**
(DOCX)

File S1 **This file includes: Table S2. Annotation of miRNAs with greater than 5 fold changes, as shown in** **Figure 2** **; Table S3. BMMC related molecules potentially targeted by the miRNAs; and Table S4. BMMC related canonical pathways potentially regulated by the miRNAs.**
(XLS)

File S2 **This file includes: Table S5: Potential binding sites between cKit and FcεRIα and their respective miRNAs; Table S6: Potential binding sites between Mitf, c/EBPα and Gata1, and their respective miRNAs; Table S7: Potential binding sites between Stat3, Stat5a, Stat5b, Gata3 and Gata2, and their respective miRNAs; and Table S8: Potential binding sites between Ndst2, mMCP4, mMCP6 and MPO, and their respective miRNAs.**
(DOCX)

Table S1 **Primer sequence for determining mRNA levels by quantitative PCR.**
(DOC)

## References

[1] Abraham SN, St John AL (2010) Mast cell-orchestrated immunity to pathogens. Nat Rev Immunol 10: 440–452. 20498670 10.1038/nri2782 PMC4469150

[2] Garcia-Montero AC, Jara-Acevedo M, Teodosio C, Sanchez ML, Nunez R, et al (2006) KIT mutation in mast cells and other bone marrow hematopoietic cell lineages in systemic mast cell disorders: a prospective study of the Spanish Network on Mastocytosis (REMA) in a series of 113 patients. Blood 108: 2366–2372. 16741248 10.1182/blood-2006-04-015545

[3] Alvarez-Twose I, Gonzalez de Olano D, Sanchez-Munoz L, Matito A, Esteban-Lopez MI, et al. (2010) Clinical, biological, and molecular characteristics of clonal mast cell disorders presenting with systemic mast cell activation symptoms. J Allergy Clin Immunol 125: : 1269–1278 e1262. 10.1016/j.jaci.2010.02.019 20434205

[4] Travis WD, Li CY, Su WP (1985) Adult-onset urticaria pigmentosa and systemic mast cell disease. Am J Clin Pathol 84: 710–714. 4072966 10.1093/ajcp/84.6.710

[5] Dawicki W, Marshall JS (2007) New and emerging roles for mast cells in host defence. Curr Opin Immunol 19: 31–38. 17126541 10.1016/j.coi.2006.11.006

[6] Hofmann AM, Abraham SN (2009) New roles for mast cells in modulating allergic reactions and immunity against pathogens. Curr Opin Immunol 21: 679–686. 19828301 10.1016/j.coi.2009.09.007 PMC2787974

[7] Galli SJ, Nakae S, Tsai M (2005) Mast cells in the development of adaptive immune responses. Nat Immunol 6: 135–142. 15662442 10.1038/ni1158

[8] Kawakami T, Galli SJ (2002) Regulation of mast-cell and basophil function and survival by IgE. Nat Rev Immunol 2: 773–786. 12360215 10.1038/nri914

[9] Mitsui H, Furitsu T, Dvorak AM, Irani AM, Schwartz LB, et al (1993) Development of human mast cells from umbilical cord blood cells by recombinant human and murine c-kit ligand. Proc Natl Acad Sci U S A 90: 735–739. 7678463 10.1073/pnas.90.2.735 PMC45740

[10] Lantz CS, Boesiger J, Song CH, Mach N, Kobayashi T, et al (1998) Role for interleukin-3 in mast-cell and basophil development and in immunity to parasites. Nature 392: 90–93. 9510253 10.1038/32190

[11] Tsuji K, Zsebo KM, Ogawa M (1991) Murine mast cell colony formation supported by IL-3, IL-4, and recombinant rat stem cell factor, ligand for c-kit. J Cell Physiol 148: 362–369. 1717495 10.1002/jcp.1041480306

[12] Kitamura Y, Go S, Hatanaka K (1978) Decrease of mast cells in W/Wv mice and their increase by bone marrow transplantation. Blood 52: 447–452. 352443

[13] Grimbaldeston MA, Chen CC, Piliponsky AM, Tsai M, Tam SY, et al (2005) Mast cell-deficient W-sash c-kit mutant Kit W-sh/W-sh mice as a model for investigating mast cell biology in vivo. Am J Pathol 167: 835–848. 16127161 10.1016/S0002-9440(10)62055-X PMC1698741

[14] Nagata H, Worobec AS, Oh CK, Chowdhury BA, Tannenbaum S, et al (1995) Identification of a point mutation in the catalytic domain of the protooncogene c-kit in peripheral blood mononuclear cells of patients who have mastocytosis with an associated hematologic disorder. Proc Natl Acad Sci U S A 92: 10560–10564. 7479840 10.1073/pnas.92.23.10560 PMC40651

[15] Arinobu Y, Iwasaki H, Gurish MF, Mizuno S, Shigematsu H, et al (2005) Developmental checkpoints of the basophil/mast cell lineages in adult murine hematopoiesis. Proc Natl Acad Sci U S A 102: 18105–18110. 16330751 10.1073/pnas.0509148102 PMC1312421

[16] Iwasaki H, Mizuno S, Arinobu Y, Ozawa H, Mori Y, et al (2006) The order of expression of transcription factors directs hierarchical specification of hematopoietic lineages. Genes Dev 20: 3010–3021. 17079688 10.1101/gad.1493506 PMC1620021

[17] Takemoto CM, Lee YN, Jegga AG, Zablocki D, Brandal S, et al (2008) Mast cell transcriptional networks. Blood Cells Mol Dis 41: 82–90. 18406636 10.1016/j.bcmd.2008.02.005 PMC2478671

[18] Gilfillan AM, Tkaczyk C (2006) Integrated signalling pathways for mast-cell activation. Nat Rev Immunol 6: 218–230. 16470226 10.1038/nri1782

[19] Mayoral RJ, Pipkin ME, Pachkov M, van Nimwegen E, Rao A, et al (2009) MicroRNA-221-222 regulate the cell cycle in mast cells. J Immunol 182: 433–445. 19109175 10.4049/jimmunol.182.1.433 PMC2610349

[20] Baltimore D, Boldin MP, O'Connell RM, Rao DS, Taganov KD (2008) MicroRNAs: new regulators of immune cell development and function. Nat Immunol 9: 839–845. 18645592 10.1038/ni.f.209

[21] Yang M, Mattes J (2008) Discovery, biology and therapeutic potential of RNA interference, microRNA and antagomirs. Pharmacol Ther 117: 94–104. 17928059 10.1016/j.pharmthera.2007.08.004

[22] Foster PS, Plank M, Collison A, Tay HL, Kaiko GE, et al (2013) The emerging role of microRNAs in regulating immune and inflammatory responses in the lung. Immunol Rev 253: 198–215. 23550648 10.1111/imr.12058

[23] Gurish MF, Ghildyal N, McNeil HP, Austen KF, Gillis S, et al (1992) Differential expression of secretory granule proteases in mouse mast cells exposed to interleukin 3 and c-kit ligand. J Exp Med 175: 1003–1012. 1372640 10.1084/jem.175.4.1003 PMC2119178

[24] Mattes J, Collison A, Plank M, Phipps S, Foster PS (2009) Antagonism of microRNA-126 suppresses the effector function of TH2 cells and the development of allergic airways disease. Proc Natl Acad Sci U S A 106: 18704–18709. 19843690 10.1073/pnas.0905063106 PMC2773983

[25] Johnnidis JB, Harris MH, Wheeler RT, Stehling-Sun S, Lam MH, et al (2008) Regulation of progenitor cell proliferation and granulocyte function by microRNA-223. Nature 451: 1125–1129. 18278031 10.1038/nature06607

[26] Larsen MT, Hother C, Hager M, Pedersen CC, Theilgaard-Monch K, et al (2013) MicroRNA profiling in human neutrophils during bone marrow granulopoiesis and in vivo exudation. PLoS One 8: e58454. 23554893 10.1371/journal.pone.0058454 PMC3595296

[27] Gerrits A, Walasek MA, Olthof S, Weersing E, Ritsema M, et al (2012) Genetic screen identifies microRNA cluster 99b/let-7e/125a as a regulator of primitive hematopoietic cells. Blood 119: 377–387. 22123844 10.1182/blood-2011-01-331686

[28] Dostalova Merkerova M, Krejcik Z, Votavova H, Belickova M, Vasikova A, et al (2011) Distinctive microRNA expression profiles in CD34+ bone marrow cells from patients with myelodysplastic syndrome. Eur J Hum Genet 19: 313–319. 21150891 10.1038/ejhg.2010.209 PMC3061996

[29] Lee ST, Chu K, Im WS, Yoon HJ, Im JY, et al (2011) Altered microRNA regulation in Huntington's disease models. Exp Neurol 227: 172–179. 21035445 10.1016/j.expneurol.2010.10.012

[30] Ma F, Liu X, Li D, Wang P, Li N, et al (2010) MicroRNA-466l upregulates IL-10 expression in TLR-triggered macrophages by antagonizing RNA-binding protein tristetraprolin-mediated IL-10 mRNA degradation. J Immunol 184: 6053–6059. 20410487 10.4049/jimmunol.0902308

[31] Lewis BP, Burge CB, Bartel DP (2005) Conserved seed pairing, often flanked by adenosines, indicates that thousands of human genes are microRNA targets. Cell 120: 15–20. 15652477 10.1016/j.cell.2004.12.035

[32] Garcia DM, Baek D, Shin C, Bell GW, Grimson A, et al (2011) Weak seed-pairing stability and high target-site abundance decrease the proficiency of lsy-6 and other microRNAs. Nat Struct Mol Biol 18: 1139–1146. 21909094 10.1038/nsmb.2115 PMC3190056

[33] Furumoto Y, Charles N, Olivera A, Leung WH, Dillahunt S, et al (2011) PTEN deficiency in mast cells causes a mastocytosis-like proliferative disease that heightens allergic responses and vascular permeability. Blood 118: 5466–5475. 21926349 10.1182/blood-2010-09-309955 PMC3217349

[34] Sakai H, Toyota N, Ito F, Takahashi H, Hashimoto Y, et al (1999) Glucocorticoids inhibit proliferation and adhesion of the IL-3-dependent mast cell line, MC/9, to NIH/3T3 fibroblasts, with an accompanying decrease in IL-3 receptor expression. Arch Dermatol Res 291: 224–231. 10335920 10.1007/s004030050398

[35] Ma P, Mali RS, Munugalavadla V, Krishnan S, Ramdas B, et al (2011) The PI3K pathway drives the maturation of mast cells via microphthalmia transcription factor. Blood 118: 3459–3469. 21791431 10.1182/blood-2011-04-351809 PMC3186328

[36] Bischoff SC (2007) Role of mast cells in allergic and non-allergic immune responses: comparison of human and murine data. Nat Rev Immunol 7: 93–104. 17259966 10.1038/nri2018

[37] Marshall JS (2004) Mast-cell responses to pathogens. Nat Rev Immunol 4: 787–799. 15459670 10.1038/nri1460

[38] Baumann H, Gauldie J (1994) The acute phase response. Immunol Today 15: 74–80. 7512342 10.1016/0167-5699(94)90137-6

[39] Maruotti N, Crivellato E, Cantatore FP, Vacca A, Ribatti D (2007) Mast cells in rheumatoid arthritis. Clin Rheumatol 26: 1–4. 16741781 10.1007/s10067-006-0305-3

[40] Duelli A, Ronnberg E, Waern I, Ringvall M, Kolset SO, et al (2009) Mast cell differentiation and activation is closely linked to expression of genes coding for the serglycin proteoglycan core protein and a distinct set of chondroitin sulfate and heparin sulfotransferases. J Immunol 183: 7073–7083. 19915053 10.4049/jimmunol.0900309

[41] Rusca N, Deho L, Montagner S, Zielinski CE, Sica A, et al (2012) MiR-146a and NF-kappaB1 regulate mast cell survival and T lymphocyte differentiation. Mol Cell Biol 32: 4432–4444. 22927641 10.1128/MCB.00824-12 PMC3486148

[42] Molnar V, Ersek B, Wiener Z, Tombol Z, Szabo PM, et al (2012) MicroRNA-132 targets HB-EGF upon IgE-mediated activation in murine and human mast cells. Cell Mol Life Sci 69: 793–808. 21853268 10.1007/s00018-011-0786-3 PMC11114963

[43] Mayoral RJ, Deho L, Rusca N, Bartonicek N, Saini HK, et al (2011) MiR-221 influences effector functions and actin cytoskeleton in mast cells. PLoS One 6: e26133. 22022537 10.1371/journal.pone.0026133 PMC3192147

[44] Monticelli S, Ansel KM, Xiao C, Socci ND, Krichevsky AM, et al (2005) MicroRNA profiling of the murine hematopoietic system. Genome Biol 6: R71. 16086853 10.1186/gb-2005-6-8-r71 PMC1273638

[45] Lee YN, Brandal S, Noel P, Wentzel E, Mendell JT, et al (2011) KIT signaling regulates MITF expression through miRNAs in normal and malignant mast cell proliferation. Blood 117: 3629–3640. 21273305 10.1182/blood-2010-07-293548 PMC3072881

[46] Siemens H, Jackstadt R, Kaller M, Hermeking H (2013) Repression of c-Kit by p53 is mediated by miR-34 and is associated with reduced chemoresistance, migration and stemness. Oncotarget 4: 1399–1415. 24009080 10.18632/oncotarget.1202 PMC3824539

[47] Shin J, Pan H, Zhong XP (2012) Regulation of mast cell survival and function by tuberous sclerosis complex 1. Blood 119: 3306–3314. 22362037 10.1182/blood-2011-05-353342 PMC3321856

