# Supplementary material for: Identification of MicroRNAs Regulating the Developmental Pathways of Bone Marrow Derived Mast Cells
Source: PLoS One. 2014 May 21;9(5):e98139. doi: 10.1371/journal.pone.0098139 (PMC4029961; doi:10.1371/journal.pone.0098139)
Supplement: Method S1 — Toluidine blue staining, flow cytometry and miRNA/mRNA qPCR were described. (DOCX) [file pone.0098139.s001.docx]

**Supplementary Methods**

**Toluidine blue staining**

1×10^6^ BMMC were centrifuged in 100μl aliquots onto clean glass slides for 5 min at 300× g using a Cytospin centrifuge. Cytospin preparations were air dried and fixed in Mota’s fixative (25ml 100% ethanol, 2 g lead acetate (Sigma-Aldrich), 1 ml glacial acetic acid, 25 ml distilled H_2_O) followed by staining with 0.5% acid toluidine blue (Sigma-Aldrich). Mast cells contain granules within their cytoplasm that exhibit blue metachromasia, after staining with acid toluidine blue.

**Flow cytometry**

Cells (3×10^5^) were incubated first with mouse F_c_ Block (2.4G2; BD PharMingen, San Diego, CA, USA) to inhibit nonspecific binding of antibodies. After washing, cells were stained with anti-c-Kit and anti-FcεRI antibodies (BD Biosciences and PharMingen, respectively). Numbers of positive cells were quantified by flow cytometry (FACSCanto flow cytometer, BD Biosciences, San Jose, CA). MCs were categorized as c-Kit^+^ FcεRI^+^ cells.[^24^](#_ENREF_24) Data was analysed with FlowJo software (version 10, Tree Star, Inc.).

**MicroRNA quantitative polymerase chain reaction**

MicroRNA quantitative polymerase chain reactions (qPCR) were performed using the TaqMan MicroRNA reverse transcription kit (Life Technologies), Taqman MicroRNA qPCR assays (Life Technologies) and TaqMan Universal PCR Master Mix, as previously described.^24^ Quantitative PCR reactions were performed according to the manufacturer’s suggested conditions. Sno202 was used as a housekeeping control RNA in the experiments. Relative expression was calculated using the 2^-ΔΔCt^ method.

**Messenger RNA qPCR**

The method for qPCR has been described in detail elsewhere.[^24^](#_ENREF_24) Briefly, total RNA was isolated from bone marrow cells and BMMC culture from week 2 to week 6 with TriReagent (Sigma-Aldrich) and reverse transcribed using M-MLV reverse transcriptase (Invitrogen). qPCR was performed using an Applied Biosystems Viia7 (Applied Biosystems, Carlsbad, CA). Amplicons were confirmed using SYBR green and expression was normalized to hypoxanthine-guanine phosphoribosyl transferase (HPRT). Primer sequences are shown in Supplementary Table 1.
