# Supplementary material for: Identification of MicroRNAs Regulating the Developmental Pathways of Bone Marrow Derived Mast Cells
Source: PLoS One. 2014 May 21;9(5):e98139. doi: 10.1371/journal.pone.0098139 (PMC4029961; doi:10.1371/journal.pone.0098139)
Supplement: File S2 — This file includes: Table S5: Potential binding sites between cKit and FcεRIα and their respective miRNAs; Table S6: Potential binding sites between Mitf, c/EBPα and Gata1, and their respective miRNAs; Table S7: Potential binding sites between Stat3, Stat5a, Stat5b, Gata3 and Gata2, and their respective miRNAs; and Table S8: Potential binding sites between Ndst2, mMCP4, mMCP6 and MPO, and their respective miRNAs. (DOCX) [file pone.0098139.s003.docx]

**Table S5: Potential binding sites between cKit and FcεRI**

**and their respective miRNAs**

| **Target Gene** | **microRNAs** | **Binding Sites** |
| --- | --- | --- |
| **cKit,**  **NM_021099** | miR-130a | 3' uacgggaaAAUUGU-AACGUGAc 5'   \| \| \|: \|\|\|\|\|\|\|  1640:5' ggaagaagUGAUCGUUUGCACUu 3' |
|  | miR-218 | 3' uguaccaAUC-UAGUUCGUGUu 5'   \|\|\| \| :\|\|\|\|\|\|  1059:5' ucucacaUAGCAGGGAGCACAa 3' |
|  |  | 3' ugUACCAAUCUAGUUCGUGUu 5'   \|\|\| \|\| : :\|\|\|\|\|\|\|   889:5' aaAUGUAUAUGCUAAGCACAa 3' |
|  |  | 3' uguaccaaucuaGUUCGUGUu 5'  \|:\|\|\|\|\|\|   141:5' auuccgccuuuaCGAGCACAc 3' |
|  | miR-223 | 3' acCCCAUAAAC-UGUUUGACUGu 5'  \|\|\| \| \|\| \|\| \|\|\|\|\|\|\|   610:5' ucGGGCUUCUGUAC-AACUGACc 3' |
|  | miR-421 | 3' cgcGGGUUA-AUUACA----GACAACUa 5'   :\|::\|\| \|: \|\|\| \|\|\|\|\|\|\|  2101:5' uuuUCUGAUAUGUUGUCCAACUGUUGAc 3' |
|  |  | 3' cgcgGGUUAAUUAC---AGAC-AACUa 5'   \|\|\|:\|\|\| \| \|\|\|\| \|\|\|\|  1730:5' gucaCCAGUUACCGCGCUCUGUUUGAa 3' |
|  | miR-505 | 3' ucuuuuggucGUUCACAACUGc 5'   \|\|\| \|\|\|\|\|\|\|  2108:5' auauguugucCAACUGUUGACa 3' |
|  | miR-542-3p | 3' aaagUCAAUAGUUAGACAGUGu 5'   \|\|\|\| \| \|\| \|\|\|\|\|\|  2067:5' ugcaAGUU-UAAACAUGUCACg 3' |
|  |  | 3' aaAGUCAAUAGUUAGACAGUGu 5'   \|\|\| \|\| \|: \|\|\|\|\|\|\|  1714:5' ugUCAUGUACGAGACUGUCACc 3' |
|  |  | 3' aaagucaAUAGUU--AGACAGUGu 5'   \|\| \|\|: \|\|\|\|\|\|\|\|  1228:5' caagacgUAACAGCUUCUGUCACc 3' |
| FcεRI,  NM_010184 | miR-363 | 3' auGUCUACC----UAUGGCACGUUAa 5'  \|\|\| \|\|\| :\|\| \| \|\|\|\|\|\|   156:5' ugCAGCUGGUUAUGUAACAUGCAAUa 3' |

**Table S6: Potential binding sites between Mitf, c/EBPα and Gata1, and their respective miRNAs**

| **Target Gene** | **microRNAs** | **Binding Sites** |
| --- | --- | --- |
| Mitf,  NM_008601 | miR-181d | 3' uggguggcuguuguuaCUUACAa 5'   \|\|\|\|\|\|   371:5' uaaaguuuaauuacacGAAUGUa 3' |
|  | miR-1896 | 3' gagGAGUGGGUGGUAGUCUCUc 5'   :\|: ::::\|: \|\|\|\|\|\|\|   270:5' ggaUUUUUUUGCU-UCAGAGAa 3' |
|  |  | 3' gaGGAGUGGGUGGUAGU-CUCUc 5'   :\| \| \|:\| :\| \|\|\| \|\|\|\|   33:5' gcUCCCUCUCUUCUUCAGGAGAc 3' |
|  | miR-207 | 3' cucccuccUCUCGGUCCUCUUCg 5'  \|\|\| \|\|\|\|\|\|\|\|   428:5' auuaugauAGAAGAAGGAGAAGa 3' |
|  |  | 3' cucccuccucucggucCUCUUCg 5'  \|\|\|\|\|\|   270:5' ggauuuuuuugcuucaGAGAAGu 3' |
|  | miR-342-3p | 3' ugcCCACGCUAAAGACACACUCu 5'  \|\| \| \| \|: \|\|\|\|\|\|\|   313:5' ucaGGGGAAAACUUGGUGUGAGc 3' |
|  |  | 3' ugcccacGCUAAAGA----CACACUCu 5'  :\|\|\|\|\|\|\| \|\|\|\|\|\|\|   230:5' ugugaacUGAUUUCUCCAAGUGUGAGc 3' |
|  |  | 3' ugcccacgcuaaagacACACUCu 5'  \|\|\|\|\|\|   139:5' uuuauuuuuagaauuuUGUGAGc 3' |
|  | miR-710 | 3' gagUUGAGAGG--GGUUCUGAACc 5'  \|\|:\|:\| : \| \|\|\|\|\|\|\|   147:5' uagAAUUUUGUGAGCCAGACUUGu 3' |
|  | miR-298 | 3' cccuucuuguCGGGA-GGAGACGg 5'  \|\|:\|\| \|\|\|\|\|\|\|   3:5' agccugccuuGCUCUGCCUCUGCa 3' |
| c/EBPα  NM_007678 | miR-1894-3p | 3' gagggaaGUGGGAGAGGGAACg 5'   \| \|\|: \|\|\|\|\|\|\|   562:5' uggagggCUCCUAAUCCCUUGc 3' |
|  | miR-218 | 3' uguaccaaucuAGUUCGUGUu 5'   \| \|\|\|\|\|\|\|   819:5' cugaaggaacuUGAAGCACAa 3' |
|  | miR-330 | 3' cggauucuguguccGGGUCUCu 5'   \|\|\|\|\|\|\|  1109:5' uuuuucgucugccuCCCAGAGg 3' |
|  |  | 3' cggauuCUGUGUCCGGGUCUCu 5'  \|\|: \|\| \|\|\|\|\|\|\|   836:5' acaaucGAUCCA-UCCCAGAGg 3' |
|  |  | 3' cgGAUUCUGUGUCCGGGUCUCu 5'  \|\| :\| :: \|\| \|\|\|\|\|\|\|   34:5' ccCU-GGCUGGAGACCCAGAGg 3' |
|  | miR-672 | 3' agUGUGUGUCAUGUGGUUGGAGu 5'  \|:\|:\| \| \|:\|:\|\|\| \|\|\|  1408:5' uaAUAUA-AACAUAUCAAACUCa 3' |
|  | miR-671-5p | 3' gaggucggggagguccCGAAGGa 5'  \|\|\|\|\|\|  1319:5' ggagcagggcuggaccGCUUCCu 3' |
|  |  | 3' gaGGUCGG-GGAGGUCCCGAAGGa 5'  \|\|:\|\|: \|: \|\|\|\|\|\|\|  1205:5' cuCCGGCUAAGACUUAGGCUUCCc 3' |
|  |  | 3' gaggucggggagguccCGAAGGa 5'  \|\|\|\|\|\|  1184:5' guaguagugggucuuaGCUUCCu 3' |
|  |  | 3' gaggucggggagguCCCGAAGGa 5'  \|\|\|\|\|\|\|\|  1133:5' caaugaaaugaaguGGGCUUCCc 3' |
|  |  | 3' gaggucggggagguccCGAAGGa 5'  \|\|\|\|\|\|   858:5' acuggaguuaugacaaGCUUCCc 3' |
|  |  | 3' gaggucggggagguccCGAAGGa 5'  \|\|\|\|\|\|   360:5' uauuuggaggauuccuGCUUCCu 3' |
|  | miR-129-3p | 3' uacgaaaaACCCCAUUCCCGAa 5'  \|\|\|\| \|\|\|\|\|\|  1309:5' ccuguggcUGGGAGCAGGGCUg 3' |
|  |  | 3' uacgaaaaACCCCAUUCC-CGAa 5'  \|\|\|\|\| \|\|\|\| \|\|\|  1046:5' cuaacggcUGGGGGAAGGAGCUg 3' |
|  |  | 3' uaCGAAAAACC-CCA-UUCCCGAa 5'  \|\|\| \|\| \|\|\| :\|\|\|\|\|\|   548:5' caGCUACAGGGAGGUGGAGGGCUc 3' |
| Gata1,  NM_008089 | miR-290-5p | 3' uuucacggggguaucAAACUCa 5'  \|\|\|\|\|\|   208:5' guuuguuguuguuguUUUGAGa 3' |
|  | miR-196b | 3' ggguuguuguccuuuGAUGGAu 5'   \|\|\|\|\|\|   116:5' cccauucuccugccuCUACCUc 3' |
|  | let-7e | 3' uuGAUAUGUUGGAGGAUGGAGu 5'  :\| \| \| :\|\|\| \|\|\|\|\|\|\|   118:5' caUUCUCCUGCCU-CUACCUCc 3' |
|  | miR-200b | 3' aguagUAAUGGUCCGU-CAUAAu 5'  \|\|\| \|\|\|\|\|\| \|\|\|\|\|   145:5' cugagAUU--CAGGCAUGUAUUg 3' |
|  | miR-139-5p | 3' gaCCUCUGUGC-ACGUGACAUCu 5'  \|\|\|\|\|\|\| \| \| : \|\|\|\|\|\|   51:5' uuGGAGACAGGAUCUUCUGUAGc 3' |

**Table S7: Potential binding sites between Stat3, Stat5a, Stat5b, Gata3 and Gata2, and their respective miRNAs**

| **Target Gene** | **microRNAs** | **Binding Sites** |
| --- | --- | --- |
| Stat3,  NM_213659 | miR-874 | 3' agccagggagcccggUCCCGUc 5'  \|\|\|\|\|\|  1361:5' ccagugucugaauuaAGGGCAg 3' |
|  |  | 3' agccagggagcccgGUCCCGUc 5'  \|\|\|\|\|\|\|  1055:5' gauaucuguaaccaCAGGGCAa 3' |
|  |  | 3' agccagggAGCCCGGUCCCGUc 5'  \| \|\| \| \|\|\|\|\|\|   726:5' gugugaggUAGGCCAAGGGCAc 3' |
|  |  | 3' agccagggagcccggUCCCGUc 5'  \|\|\|\|\|\|   479:5' aaaaauaagaauuaaAGGGCAa 3' |
|  | miR-101a | 3' aagucAAUAGUG-UCAUGACAu 5'  \|\|:\|\| : \| \|\|\|\|\|\|   105:5' ggguuUUGUCUUGAAUACUGUu 3' |
|  | miR-330 | 3' cgGAUUCUGUGUCCGGGUCUCu 5'  \|\|\|:\|\|\| :\| \|\|\|\|\|\|\|\|   435:5' agCUAGGAC-UAAGCCCAGAGg 3' |
|  | miR-125b-5p | 3' agUGUUCA-AUCCCAGAGUCCCu 5'  :\| :\|\| \| \| \|\|\|\|\|\|\|  1591:5' ggGCUGGUGUUGUACCUCAGGGg 3' |
|  |  | 3' aguguucaaucccaGAGUCCCu 5'  \|\|\|\|\|\|\|   999:5' ggcccucagcaaagCUCAGGGa 3' |
|  |  | 3' aguguucaauccCAGAGUCCCu 5'  \|\|:\|\|\|\|\|\|   789:5' acuccagacccuGUUUCAGGGu 3' |
| Stat5a,  NM_011488 | miR-342-5p | 3' ugcccacgcuaAAGACACACUCu 5'  \|\|\| \|\|\|\|\|\|\|   24:5' ggaaaaccaccUUCAGUGUGAGg 3' |
| Stat5b,  NM_011489 | miR-23b | 3' ccaUUAGGGACCGUUACACUa 5'  \|\|\| \|\| \|:\|\|\|\|\|\|\|   86:5' aacAAU-ACUCGUAAUGUGAa 3' |
|  | miR-342-3p | 3' ugcccacgcUAAAGACACA-CUCu 5'  \|\|\|\|: \|\|\|\| \|\|\|  2241:5' cagauacauAUUUUAGUGUCGAGg 3' |
|  |  | 3' ugcccacgcuaaagaCACACUCu 5'  \|\|\|\|\|\|\|  1662:5' ggccggauggaggagGUGUGAGg 3' |
|  |  | 3' ugCCCACGCUAAAGACACACUCu 5'  \| \|\|\|\|\| \| \|\|\|\|\|\|\|   159:5' ggGAGUGCG-CGUGAGUGUGAGu 3' |
|  | miR-125b-5p | 3' aguguucaaucccagAGUCCCu 5'  \|\|\|\|\|\|  1327:5' ggcugcagugggccgUCAGGGa 3' |
|  |  | 3' agugUUCAAUCCCAGAGUCCCu 5'  \|\|\|\| \| \| \|\|\|\|\|\|\|  1258:5' agcaAAGUGAAAGCCUCAGGGu 3' |
|  |  | 3' aguguucaaucccagAGUCCCu 5'  \|\|\|\|\|\|   785:5' ccuguguuccuucugUCAGGGc 3' |
|  |  | 3' aguguucaauCCCA-GAGUCCCu 5'  \| \|\| :\|\|\|\|\|\|   302:5' accuggccauGUGUCUUCAGGGu 3' |
|  | miR-132 | 3' gcugguaccgacaucUGACAAu 5'  \|\|\|\|\|\|  1512:5' auuuaucaguguuuuACUGUUa 3' |
| Gata3  NM_008091 | miR-34a | 3' uguUGGUCGAUUCUGUGACGGu 5'  \|::\|\| \|:\| \|\|\|\|\|\|\|  1211:5' uguAUUAG-UGA-UCACUGCCu 3' |
|  | miR-290-5p | 3' uuucacggggguaucAAACUCa 5'  \|\|\|\|\|\|   310:5' aagaaauacuguacaUUUGAGg 3' |
|  | miR-132 | 3' gcUGGUACCGACAUCUGACAAu 5'  \|\|\| \|\|\|\|\| \|\|\|\|\|\|\|  1179:5' ccACC-UGGCU---GACUGUUa 3' |
|  | miR-135a | 3' aguGUAUCCUUAUUUUUCGGUAu 5'  \|\|\| \| : \|\| \|\|\|\|\|\|\|   208:5' uccCAUUUGUG-AAUAAGCCAUu 3' |
| Gata2  NM_008090 | miR-200b | 3' aguaguaaUGGUCCGUCAUAAu 5'  \|:\| \|\|\|\|\|\|\|\|\|  1361:5' agagaagaAUC-GGCAGUAUUu 3' |
|  | miR-363 | 3' auguCUACCUA--UGG--CACGUUAa 5'  \| \|\| :\| \|\|\| \|\|\|\|\|\|\|  1270:5' uuguGCUGAGUCAACCAAGUGCAAUa 3' |
|  | miR-132 | 3' gcugguaccgacaucUGACAAu 5'  \|\|\|\|\|\|  1492:5' uccuuuaaagugaauACUGUUa 3' |

**Table S8: Potential binding sites between Ndst2, mMCP4, mMCP6 and MPO, and their respective miRNAs**

| **Target Gene** | **microRNAs** | **Binding Sites** |
| --- | --- | --- |
| Ndst2,  NM_010811 | miR-671-5p | 3' gaggucggggaggUCCCGAAGGa 5'  :\|\|\|\|\|\|\|\|   169:5' gggaccuaauaaaGGGGCUUCCc 3' |
|  | miR-298 | 3' cccuucuugucGGGAGGAG-ACGg 5'  \|\|:\|\|\|\|\| \|\|\|   280:5' uucagugguuuCCUUCCUCGUGCc 3' |
|  | miR-135a | 3' agUGUAUCCUUAUUUUUCGGUAu 5'  \|\|\| \| \| \|:\|\|\|\|\|\|   430:5' agACAGUUGCUCAUAGAGCCAUa 3' |
|  |  | 3' aguguaUCCUUAUUUUUC-GGUAu 5'  \|\|\|::\|\|\|:\|\|\| \|\|\|\|   256:5' cugggaAGGGGUAAGAAGACCAUa 3' |
|  | let-7e | 3' uugauauGUUGGAGGAUGGAGu 5'  :\| \|\| :\|\|\|\|\|\|\|   87:5' ggccccuUACCCCUCUACCUCa 3' |
|  | miR-1894-3p | 3' gagggaagugggaGAGGGAACg 5'  :\|\|\|\|\|\|\|   174:5' cuaauaaaggggcUUCCCUUGg 3' |
| mMCP4,  NM_010779 | miR-132 | 3' gcuGGUACCGACAUCUGACAAu 5'  \|::\|\| : \|:\|\|\|\|\|\|\|   156:5' auuCUGUGAUGAUGGACUGUUc 3' |
|  | miR-330 | 3' cggauucuguguccgGGUCUCu 5'  \|\|\|\|\|\|   22:5' aucagagucuucaagCCAGAGc 3' |
|  | miR-501-3p | 3' guUUAGGAACGGGCCCACGUAa 5'  \|\| \|\|\| \|\|:\| \|\|\|\|\|\|   3:5' aaAAGCCUGACCUGCGUGCAUc 3' |
|  | miR-542-3p | 3' aaaGUCAAUAGUUA-GACAGUGu 5'  \|\| \|:\|\| \|\| \|\|\|\|\|\|\|   70:5' aagCAUGUGUCCAUCCUGUCACc 3' |
|  | miR-672 | 3' agUGU-GUGUCAU---GUGGUUGGAGu 5'  :\|\| \| \|\|\|\|\| \| \|:\|\|\|\|\|\|   184:5' auGCACCUCAGUAAAGCCCUAACCUCc 3' |
|  | miR-1894-3p | 3' gaGGGAAGUGGGAGAGGGAACg 5'  \|:\|\|\|\| : \| \|\|\|\|\|\|   41:5' agCUCUUC-UGAUAACCCUUGg 3' |
| mMCP6  NM_010781 | miR-705 | 3' acGGGUGGGGUGGAGGGUGg 5'   :\|\| :\|:::\|\|\|\|\|\|\|\|   166:5' guUCCCUCUUGCCUCCCACc 3' |
| MPO  NM_010824 | miR-207 | 3' cuCCCUCCUCUCGGUCCUCUUCg 5'  \|\|\|\| \| \|\|\|:\|\|\|\|\|\|\|   4:5' uuGGGA-GUCUGCCGGGAGAAGg 3' |
