# Supplementary material for: Identification of MicroRNAs Regulating the Developmental Pathways of Bone Marrow Derived Mast Cells
Source: PLoS One. 2014 May 21;9(5):e98139. doi: 10.1371/journal.pone.0098139 (PMC4029961; doi:10.1371/journal.pone.0098139)
Supplement: Table S1 — Primer sequence for determining mRNA levels by quantitative PCR. (DOC) [file pone.0098139.s004.doc]

**Table S1: Primer pairs**

| **Primer Name** | **Direction** | **Sequence (5’-3’)** |
| --- | --- | --- |
| HPRT | Forward | AGGCCAGACTTTGTTGGATTTGAA |
|  | Reverse | CAACTTGCGCTCATCTTAGGCTTT |
| cKit | Forward | CGGTCGACTCCAAGTTCTACAAG |
|  | Reverse | GTTGCAGTTTGCCAAGTTGGAGT |
| FceRIa | Forward | TGCCACCGTTCAAGACAG |
|  | Reverse | TTGCGGACATTCCAGTTC |
| IL3Rα | Forward | ACATCACGACAGGGGCTTTT |
|  | Reverse | GGGAGAGCGACTGGAATGAG |
| GATA1 | Forward | CGCTCCCTGTCACCGGCAGTGC |
|  | Reverse | CCGCCACAGTGGAGTAGCCGTT |
| GATA2 | Forward | CTCCCGACGAGGTGGATGTCTT |
|  | Reverse | CCTGGGCTGTGCAACAAGTGTG |
| GATA3 | Forward | AGAACCGGCCCCTTATGAA |
|  | Reverse | AGTTCGCGCAGGATGTCC |
| Mitf | Forward | AGCAACGAGCTAAGGACC |
|  | Reverse | GGATGGGATAAGGGAAAGT |
| Sfpi1 (PU.1) | Forward | TGTCCACAACAACGAGTTTGAGAA |
|  | Reverse | GGGACAAGGTTTGATAAGGGAAGC |
| C/EBPα | Forward | GCATCTGCGAGCACGAGACGCT |
|  | Reverse | CGCCTTGGCCTTCTCCTGCTGT |
| Stat3 | Forward | GGGCTCTTGTCAGCAATGGAGT |
|  | Reverse | TTGGCGGGTCTGAAGTTGAGAT |
| Stat5a | Forward | CCAAGTCCCTGCTCAAGAATGA |
|  | Reverse | AACTTCTCCTCCGTCACCGACT |
| Stat5b | Forward | TGGTTTGATGGCGTGATG |
|  | Reverse | GTCCCGTCTGGCTTGTTG |
| Hdc | Forward | GAGCCCGATGCTAATGAGTC |
|  | Reverse | GAGAAGTTGTCGTCCACAGGTA |
| Ndst2 | Forward | AGCTTTGTAATCTCGGATTGTAAG |
|  | Reverse | CAGAAGGAGCAACAGGGTGT |
| Mpo | Forward | CATGCGCTCCAGCGAGAT |
|  | Reverse | CAACACCAAGGGCAGGTAGTC |
| mMCP4 | Forward | TCCTCTGCCTCGTCCTTC |
|  | Reverse | GATTCTCAGTTTCACCTCCC |
| mMCP6 | Forward | GTGGGACCGCACATCAAAAG |
|  | Reverse | TCAAGCTCCAGCAGGGCAAC |
